# Feed Restriction Modifies Intestinal Microbiota-Host Mucosal Networking in Chickens Divergent in Residual Feed Intake

**DOI:** 10.1128/mSystems.00261-18

**Published:** 2019-01-29

**Authors:** Barbara U. Metzler-Zebeli, Sina-Catherine Siegerstetter, Elizabeth Magowan, Peadar G. Lawlor, Renée M. Petri, Niamh E. O´Connell, Qendrim Zebeli

**Affiliations:** a Institute of Animal Nutrition and Functional Plant Compounds, Department for Farm Animals and Veterinary Public Health, University of Veterinary Medicine Vienna, Vienna, Austria; b Agriculture Branch, Hillsborough, Agri-Food and Biosciences Institute, Belfast, Northern Ireland, United Kingdom; c Teagasc, Pig Development Department, Animal & Grassland Research & Innovation Centre, Moorepark, Ireland; d Institute for Global Food Security, Queen´s University Belfast, Belfast, Northern Ireland, United Kingdom; University of Massachusetts Dartmouth

**Keywords:** chicken, feed intake level, intestinal barrier function, intestinal microbiota, intestinal physiology, residual feed intake, visceral organs

## Abstract

Differences in chickens’ feed intake may be the underlying factor influencing feed-efficiency (FE)-associated variation in intestinal microbiota and physiology. In chickens eating the same amount of feed, quantitative feed restriction may create similar intestinal conditions and help clarify this cause-and-effect relationship. This study investigated the effect of *ad libitum* versus restrictive feeding (85% of *ad libitum*) on ileal and cecal microbiota, concentrations of short-chain fatty acids, visceral organ size, intestinal morphology, permeability, and expression of genes related to nutrient uptake, barrier function, and innate immune response in broiler chickens with divergent residual feed intake (RFI; metric for FE). On day 30 posthatch, 28 low-RFI (good FE) and 29 high-RFI (poor FE) chickens across both feeding-level groups (*n *=* *112) were selected. Supervised multigroup data integration and relevance network analyses showed that especially *Lactobacillus* (negative) in ileal digesta, *Turicibacter* (positive) in cecal digesta, and *Enterobacteriaceae* (positive) in both intestinal segments depended on chicken’s feed intake, whereas the level of *Anaerotruncus* in cecal digesta was most discriminative for high RFI. Moreover, shallower crypts and fewer goblet cells in ceca indicated host-related energy-saving mechanisms with low RFI, whereas greater tissue resistance suggested a stronger jejunal barrier function in low-RFI chickens. Values corresponding to feed intake level × RFI interactions indicated larger pancreas and lower levels of ileal and cecal short-chain fatty acids in restrictively fed high-RFI chickens than in the other 3 groups, suggesting host physiological adaptations to support greater energy and nutrient needs of high-RFI chickens compensating for the restricted feeding.

**IMPORTANCE** The impact of the FE-associated differences in feed intake on intestinal bacterial and host physiological parameters has so far not been clarified. Understanding the underlying principles is essential for the development of cost-effective strategies to improve FE in chicken production. Under conditions of quantitative feed restriction, low- and high-RFI chickens ate the same amount of feed. Therefore, this research helps in distinguishing intestinal bacterial taxa and functions that were highly reliant on feed intake from those that were associated with physiological adaptations to RFI-associated differences in host nutritional needs and intestinal nutrient availability. This work provides a background for further research to assess manipulation of the intestinal microbiota, host physiology, and FE in chickens by dietary intervention.

## Abstract (precis)

The impact of the FE-associated differences in feed intake on intestinal bacterial and host physiological parameters has so far not been clarified. Understanding the underlying principles is essential for the development of cost-effective strategies to improve FE in chicken production. Under conditions of quantitative feed restriction, low- and high-RFI chickens ate the same amount of feed. Therefore, this research helps in distinguishing intestinal bacterial taxa and functions that were highly reliant on feed intake from those that were associated with physiological adaptations to RFI-associated differences in host nutritional needs and intestinal nutrient availability. This work provides a background for further research to assess manipulation of the intestinal microbiota, host physiology, and FE in chickens by dietary intervention.

## INTRODUCTION

Understanding the mechanisms that underpin individual animal variation is mandatory for the development of effective strategies to improve feed efficiency (FE) to decrease overall production costs, preserve additional edible resources for humans, and reduce the ecological footprint of chicken rearing systems (1, 2). Typically, feed-efficient chickens have lower feed intake than chickens with low feed efficiency and show differences from those chickens in their intestinal microbiota as well as in intestinal structural and functional characteristics (2–5). From the data available, it is not clear whether this difference in the feeding behavior is the sole causative factor or whether other host-related factors may underlie the FE-associated variation in the intestinal microbiota and physiology in chickens. The feed intake level (FL) of a chicken modifies the size of the visceral organs, digesta volume, retention time, and nutrient digestion (6) and may alter the intestinal microbiota composition as reported for other livestock species (e.g., sheep) (7), which may have consequences for FE. So far, the impact of the FL on the intestinal microbial community structure and function has not been elucidated in chickens of divergent FE.

In order to study the effect of the FL, it is essential that all birds eat the same amount of feed, irrespective of FE. This may be achieved by limiting the feed intake by means of quantitative feed restriction. Feed restriction in chicken rearing is used to prevent metabolic disorders (e.g., sudden death syndrome and ascites) and to manipulate carcass composition (8). However, feed restriction has also been shown to cause intestinal microbial and digestive-physiological adaptations (9, 10) and may impair the intestinal barrier function in chickens (11). The latter finding is interesting in relation to the FE of the birds because we observed greater jejunal permeability in chickens of high FE which had a limited feed intake compared to chickens of low FE in a previous study (5). In general, enhanced mucosal permeability may facilitate the paracellular uptake of nutrients but it may also allow enteric bacteria or their toxic compounds to more easily translocate into the body, thereby triggering a stronger immune response and reducing of the growth performance of the chicken (11).

If the FL is a factor influencing FE-associated variation in the intestine, it may be possible, by imposing the same limitation on all chickens with respect to the amount of feed, to create similar intestinal conditions (i.e., substrate availability, passage rate) regardless of a given bird’s FE. Therefore, we hypothesized that restrictive feeding would produce similar intestinal bacterial profiles as well as similar results with respect to development and function of the intestinal epithelium in chickens with low feed efficiency compared to chickens with high feed efficiency. Our objective was to investigate the effect of restrictive versus *ad libitum* feeding on the ileal and cecal microbiota, visceral organ size, and intestinal morphology and permeability and expression of genes in relation to nutrient transporters, barrier function, and innate immune response in broiler chickens of divergent FE. As a metric for FE, we used the residual feed intake (RFI), with a low RFI value representing good FE and a high (positive) RFI value representing poor FE. The RFI of each chicken was determined for the experimental period from 9 to 30 days posthatch (dph). As the chickens with high and low feed efficiency were assumed to have the most highly contrasting intestinal microbiota and intestinal structure and function, alterations caused by the feed restriction might be more easily detected in those chickens. Therefore, we studied only chickens with the lowest and highest RFI values in the restrictively and *ad libitum*-fed groups.

## RESULTS

### Restrictive feeding improves feed efficiency.

Chickens with extremely low and high RFI values were selected in both FL groups to discriminate between their intestinal microbiota, structure, and function characteristics. Consequently, chickens in both FL groups had largely contrasting RFI values (see Table S1 in the supplemental material). Nevertheless, as indicated by the FL × RFI interaction (*P < *0.05), restrictive feeding decreased the RFI value of high-RFI chickens by 153 g compared to *ad libitum*-fed high-RFI chickens. Although *post hoc* comparisons showed similar trends (*P < *0.10) for the 2 groups of high-RFI males, the FL × RFI interaction did not reach significance (FL × RFI, *P = *0.171). Overall, restrictive feeding lowered the total feed intake (TFI) of chickens by 338 g on average between 9 and 30 dph compared to *ad libitum*-fed birds (*P < *0.05). Because low-RFI chickens commonly ate less than high-RFI chickens (12), the feed restriction was less severe among the low-RFI chickens (92% of *ad libitum* group) than among the high-RFI chickens (80% of *ad libitum* group).

10.1128/mSystems.00261-18.1 TABLE S1 Total feed intake (TFI), total body weight gain (TBWG), and residual feed intake (RFI) values of low- and high-RFI broiler chickens fed either *ad libitum* or restrictively. Download Table S1, PDF file, 0.05 MB. Copyright © 2019 Metzler-Zebeli et al. 2019 Metzler-Zebeli et al. This content is distributed under the terms of the Creative Commons Attribution 4.0 International license.

### Feed restriction alters bacterial microbiome in ileal and cecal digesta.

**FIG 1:**
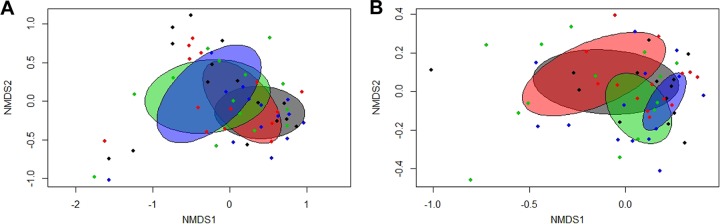
Two-dimensional nonparametric multidimensional scaling (NMDS) ordination plots of ileal (A) and cecal (B) bacterial communities of low- and high-residual-feed-intake (RFI) broiler chickens fed either *ad libitum* or restrictively. The NMDS plots were generated using Bray-Curtis distance metric data from comparisons between chicken groups. Each dot represents an individual sample; the ellipses indicate the standard deviations (SD). Blue, restrictively fed high-RFI chickens (*n *=* *7 per sex); green, restrictively fed low-RFI chickens (*n *=* *7 per sex); red, *ad libitum*-fed high-RFI chickens (*n *=* *8 females and *n *=* *7 males); gray, *ad libitum*-fed low-RFI chickens (*n *=* *7 per sex).

Samples were rarefied to 2,079 reads to account for unequal numbers of sequences between samples before calculating α-diversity indices. Restrictive feeding increased the ileal bacterial diversity (*P < *0.05) as well as the ileal species abundances (*P < *0.05) and, as a trend, cecal species abundances (*P < *0.10), whereas the RFI rank did not alter the ileal and cecal α-diversity among chicken groups (Table S2). Nonparametric multidimensional scaling (NMDS) was used to represent separation of the taxonomic composition data among the 4 groups. A significant separation was detected in Bray-Curtis-derived dissimilarity matrices (permutational multivariate analysis of variance [PERMANOVA]) for bacterial composition in ileal digesta (*P = *0.003; Fig. 1A) and cecal digesta (*P = *0.001; Fig. 1B) between feeding-level groups, whereas the RFI rank did not show a significant effect (*P > *0.10) on the β-diversity structure irrespective of the FL group examined. Moreover, it was discernible that, in particular, the taxonomic composition in the ceca of the restrictively fed high-RFI group differed from that of the *ad libitum*-fed high-RFI group, as those groups clustered separately in the NMDS plot.

10.1128/mSystems.00261-18.2 TABLE S2 Alpha-diversity of bacterial microbiota communities in ileal and cecal digesta of low- and high-residual-feed-intake (RFI) broiler chickens fed either *ad libitum* or restrictively. Download Table S2, PDF file, 0.04 MB. Copyright © 2019 Metzler-Zebeli et al. 2019 Metzler-Zebeli et al. This content is distributed under the terms of the Creative Commons Attribution 4.0 International license.

Taxonomic assessment of the bacterial microbiota in ileal and cecal digesta showed that restrictive feeding enriched the *Firmicutes* population numbers and decreased the *Proteobacteria* population by 11% at both intestinal sites (*P < *0.05; Table S3), with family-level analysis indicating certain intestinal site-specific differences in the FL-related abundances in taxa, though. In the ileal digesta, the most drastic changes in population numbers within the *Firmicutes* were among the high-abundance taxa, including a trend showing a 16.5% decrease in the population of *Turicibacteraceae* (*P = *0.058) and an 18.5% increase in the population of *Lactobacillaceae* (*P = *0.046), as well as trends for increases of 2.9% and 0.5% in the populations of *Ruminococcaceae* (*P = *0.084) and *Lachnospiraceae* (*P = *0.096), respectively, in restrictively fed chickens compared to *ad libitum*-fed chickens (Table 1). With regard to *Proteobacteria*, restrictive feeding decreased the level of *Enterobacteriaceae* by 15.7% in ileal digesta compared to *ad libitum* feeding (*P = *0.033). Similar shifts within the *Firmicutes* and *Proteobacteria* were seen in cecal digesta. In addition to the aforementioned families, populations of an unclassified family of the order *Clostridiales*, the predominant *Firmicutes* family in cecal digesta, were enriched by 13.9% in the ceca of restrictively fed birds compared to *ad libitum*-fed birds (*P < *0.001). The numbers of the second and third most dominant *Firmicutes* families in the ceca, *Ruminococcaceae* and *Lachnospiraceae,* and the dominant *Proteobacteria* family (i.e., *Enterobacteriaceae*) declined by 5.6, 3.1, and 5.0%, respectively, with restrictive compared to *ad libitum* feeding (*P < *0.05). Low-abundance *Firmicutes* families that further decreased in number with restrictive feeding included *Peptostreptococcaceae* (*P < *0.001) (and results showed similar trends for *Clostridiaceae* and *Enterococcaceae* [*P < *0.10]) in cecal digesta, whereas *Dehalobacteriaceae*, as a trend in ileal (*P = *0.054) and significantly in cecal digesta (*P = *0.007), and *Christensenellaceae*, as a trend in cecal digesta (*P < *0.10), were enriched with restrictive compared to *ad libitum* feeding. RFI-associated changes were found in the ceca only at the family level for low-abundant *Christensenellaceae* (*P < *0.05) and, as a trend, *Bacillaceae* (*P < *0.10), which were more and less abundant in low-RFI and high-RFI chickens, respectively.

**TABLE 1:** Differences in relative abundances of bacterial families present in ileal and cecal digesta of low- and high-RFI broiler chickens fed either *ad libitum* or restrictively^*a*^

Family	% Relative abundance				SEM	*P* value		
	*Ad libitum* feeding		Restrictive feeding					
	Low RFI	High RFI	Low RFI	High RFI		FL	RFI	FL × RFI
Ileum								
o__*Clostridiales*;f__	3.58	0.83	6.83	11.27	4.229	0.112	0.843	0.400
f__*Turicibacteraceae*	42.20	48.27	29.06	27.85	8.661	0.058	0.780	0.676
f__*Ruminococcaceae*	0.95	0.22	4.49	2.47	1.642	0.084	0.408	0.695
f__*Enterobacteriaceae*	37.49	22.83	17.74	11.14	7.155	0.033	0.143	0.576
f__*Lactobacillaceae*	9.38	22.13	35.34	33.30	9.064	0.046	0.557	0.419
f__*Lachnospiraceae*	0.22	0.063	0.76	0.49	0.284	0.096	0.453	0.832
f__*Peptostreptococcaceae*	3.65	3.50	2.56	6.15	1.699	0.647	0.316	0.277
f__*Clostridiaceae*	2.15	1.83	2.60	6.62	2.247	0.249	0.414	0.339
o__RF39;f__	0.023	0.008	0.060	0.071	0.033	0.134	0.949	0.700
f__*Christensenellaceae*	0.050	0.0012	0.091	0.036	0.039	0.330	0.185	0.934
f__*Bacillaceae*	0.062	0.018	0.090	0.044	0.047	0.560	0.337	0.989
o__*Streptophyta*;f__	0.13	0.10	0.23	0.18	0.086	0.289	0.665	0.912
o__*Clostridiales*; Other	0.025	0.028	0.019	0.033	0.009	0.942	0.342	0.559
f__*Dehalobacteriaceae*	0.0004	0	0.016	0.005	0.005	0.054	0.292	0.336
f__*Erysipelotrichaceae*	0.005	0.006	0.007	0.014	0.004	0.281	0.419	0.436
f__*Coriobacteriaceae*	0.113	0.100	0.090	0.060	0.019	0.103	0.258	0.673
f__*Enterococcaceae*	0.037	0.031	0.027	0.071	0.0181	0.394	0.298	0.172
f__*Corynebacteriaceae*	0.0006	0.007	0.003	0.078	0.0351	0.296	0.252	0.329

Ceca								
o__*Clostridiales*;f__	51.67	51.87	65.29	66.09	3.217	<0.001	0.876	0.926
f__*Turicibacteraceae*	0.35	0.97	0.12	0.016	0.228	0.012	0.263	0.119
f__*Ruminococcaceae*	29.00	29.54	23.37	25.23	2.358	0.040	0.614	0.781
f__*Enterobacteriaceae*	7.82	6.62	2.34	0.90	1.571	<0.001	0.406	0.940
f__*Lactobacillaceae*	0.96	0.73	1.17	1.11	0.326	0.373	0.662	0.798
f__*Lachnospiraceae*	7.94	7.65	5.24	4.16	0.978	0.003	0.487	0.688
f__*Peptostreptococcaceae*	0.062	0.048	0.022	0.010	0.011	<0.001	0.160	0.906
f__*Clostridiaceae*	0.18	0.41	0.04	0.06	0.129	0.059	0.348	0.400
o__RF39;f__	0.61	0.90	1.00	0.69	0.190	0.648	0.975	0.117
f__*Christensenellaceae*	0.52	0.17	0.65	0.49	0.123	0.079	0.047	0.427
f__*Bacillaceae*	0.38	0.42	0.19	0.76	0.179	0.661	0.095	0.142
o__*Streptophyta*;f__	0	0	0	0.0008	0.0004	0.339	0.308	0.339
o__*Clostridiales*; Other	0.072	0.106	0.104	0.078	0.031	0.938	0.887	0.343
f__*Dehalobacteriaceae*	0.084	0.079	0.135	0.136	0.019	0.007	0.938	0.863
f__*Erysipelotrichaceae*	0.086	0.165	0.072	0.073	0.033	0.113	0.226	0.243
f__*Coriobacteriaceae*	0.005	0.006	0.007	0.014	0.004	0.281	0.419	0.436
f__*Enterococcaceae*	0.005	0.005	0.002	0.003	0.003	0.069	0.753	0.615
f__*Corynebacteriaceae*	0	0.000003	0	0.0006	0.0002	0.150	0.145	0.150

a Data are presented as least-squares means and pooled standard errors of the means (SEM). *n *=* *7 per FL group, residual-feed-intake (RFI) rank, and sex except for *n *=* *8 high-RFI *ad libitum* females. RFI was calculated for the experimental period from 9 to 30 days posthatch. FL, feed intake level.

10.1128/mSystems.00261-18.3 TABLE S3 Differences in relative abundance (%) of the dominant bacterial phyla present in ileal and cecal digesta of low- and high-residual-feed-intake (RFI) broiler chickens fed either *ad libitum* or restrictively. Download Table S3, PDF file, 0.04 MB. Copyright © 2019 Metzler-Zebeli et al. 2019 Metzler-Zebeli et al. This content is distributed under the terms of the Creative Commons Attribution 4.0 International license.

### Restrictive feeding and RFI-associated effects on ileal and cecal fermentation acids.

Restrictive feeding of chickens mainly decreased the ileal and cecal concentrations of acetate, resulting in reductions in total short-chain fatty acid (SCFA) concentrations compared to *ad libitum*-fed chickens (*P < *0.05) (Table 2). In addition to the measured concentrations, FL and RFI rank affected the molar SCFA concentrations, showing that not only the acetate concentration but also its molar proportion decreased with restrictive feeding at both intestinal sites (*P < *0.05). Although RFI rank effects were observed for several single SCFAs, the FL × RFI interactions (*P < *0.05) for concentrations and molar proportions of these SCFAs showed that restrictively fed high-RFI chickens contained more valerate, isovalerate, and caproate in ileal digesta and more isobutyrate and isovalerate (only with respect to the molar proportion) in cecal digesta than the other 3 groups. Concurrently, the cecal digesta of restrictively fed low-RFI chickens was more concentrated with respect to butyrate than was seen with the other groups (*P < *0.05). ANOVA also showed higher (*P < *0.001) molar proportions of valerate in cecal digesta of restrictively fed chickens than in that of *ad libitum*-fed chickens. Moreover, the FL × RFI interaction (*P = *0.020) for propionate indicated that restrictive feeding enhanced its cecal proportion in high-RFI chickens compared to low-RFI chickens, whereas the opposite was found for *ad libitum*-fed birds.

**TABLE 2:** Differences in concentrations and molar proportions of SCFA in ileal and cecal digesta of low- and high-RFI broiler chickens fed either *ad libitum* or restrictively^*a*^

Parameter	Value^*b*^				SEM	*P* value		
	*Ad libitum* feeding		Restrictive feeding					
	Low RFI	High RFI	Low RFI	High RFI		FL	RFI	FL × RFI
Ileum								
Concn (µmol/g)								
Total SCFA	61.8	57.3	48.1	49.2	3.99	0.009	0.672	0.483
Acetate	56.1	52.2	43.5	44.0	3.61	0.006	0.628	0.549
Propionate	0.7	0.6	0.5	0.7	0.07	0.462	0.223	0.064
Butyrate	0.5	0.3	0.2	0.4	0.16	0.707	0.861	0.240
Isobutyrate	4.5	4.2	3.8	3.8	0.42	0.224	0.731	0.702
Valerate	0.05b	0.05b	0.01b	0.12a	0.02	0.405	0.024	0.033
Isovalerate	0.02b	0.02b	0b	0.07a	0.01	0.390	0.017	0.026
Caproate	0.09b	0.07b	0.05b	0.17a	0.03	0.202	0.062	0.008
Molar proportions (%)								
Acetate	90.8	91.1	90.7	89.3	0.45	0.034	0.191	0.055
Propionate	1.1	1.1	1.0	1.4	0.11	0.269	0.060	0.079
Butyrate	0.63	0.39	0.48	0.73	0.21	0.647	0.992	0.245
Isobutyrate	7.2	7.2	7.6	7.9	0.51	0.281	0.846	0.767
Valerate	0.06b	0.08b	0.04b	0.23a	0.04	0.154	0.014	0.040
Isovalerate	0.03b	0.04b	0b	0.12a	0.02	0.184	0.003	0.014
Caproate	0.15b	0.12b	0.12b	0.35a	0.05	0.048	0.063	0.012

Ceca								
Concn (µmol/g)								
Total SCFA	160.4	154.6	140.6	118.5	13.29	0.046	0.304	0.544
Acetate	131.9	127.4	109.9	93.4	11.30	0.020	0.362	0.600
Propionate	8.4	6.4	6.7	7.2	1.06	0.682	0.457	0.247
Butyrate	15.6b	16.3b	19.7a	11.8b	1.76	0.898	0.050	0.020
Isobutyrate	1.0b	1.3b	1.0b	2.2a	0.18	0.023	<0.001	0.025
Valerate	1.6	1.3	1.9	1.6	0.20	0.128	0.220	0.878
Isovalerate	1.1	1.3	1.0	1.7	0.21	0.436	0.031	0.183
Caproate	0.8	0.6	0.5	0.6	0.16	0.224	0.914	0.413
Molar proportions (%)								
Acetate	82.2	82.5	77.6	79.4	1.21	0.003	0.413	0.546
Propionate	5.3a	4.0b	4.7ab	5.8a	0.50	0.255	0.769	0.020
Butyrate	9.7b	10.6b	14.5a	9.4b	1.04	0.090	0.054	0.006
Isobutyrate	0.7b	0.9b	0.8b	2.1a	0.19	0.003	<0.001	0.009
Valerate	1.0	0.8	1.4	1.4	0.11	<0.001	0.539	0.554
Isovalerate	0.7b	0.9b	0.7b	1.6a	0.15	0.024	0.003	0.046
Caproate	0.5	0.4	0.3	0.50	0.09	0.930	0.676	0.110

a Data are presented as least-squares means and pooled SEM. *n *=* *7 per FL group, residual-feed-intake (RFI) rank, and sex except for *n *=* *8 high-RFI *ad libitum* females. RFI was calculated for the experimental period from 9 to 30 days posthatch. FL, feed intake level; SCFA, short-chain fatty acids.

b Different letters within a row indicate significant differences (*P *≤* *0.05).

### Restrictive feeding and RFI-associated differences in visceral organ size and intestinal structure and function.

Although the body weight (BW) values measured at sampling between 33 and 37 dph were not different among the 4 groups, restrictively fed chickens had heavier (*P < *0.05) crop and duodenum than *ad libitum*-fed birds and tended (*P < *0.10) to have heavier ceca (Table S4). Along with the higher weight, the duodenum was 0.9 cm longer in restrictively fed chickens than in *ad libitum*-fed chickens (*P < *0.05). Low-RFI chickens were further characterized by trends (*P < *0.10) toward lighter liver and pancreas but heavier ceca than were measured for high-RFI chickens. The FL × RFI interaction (*P < *0.01) for the pancreas weight data indicated that the RFI-associated difference existed only in restrictively fed chickens. Most histomorphological parameters in the jejunum and ileum were unaffected by FL and RFI rank (Table S5). The feed restriction tended (*P < *0.10) to decrease the ileal and cecal lymphocyte counts by 17.5% and 21.4%, respectively, compared to *ad libitum* feeding. In addition, low-RFI chickens had shallower crypts and fewer goblet cells (*P < *0.01) than high-RFI chickens and tended to have thinner longitudinal muscles (*P < *0.10) in the cecum.

10.1128/mSystems.00261-18.4 TABLE S4 Differences in body weight at sampling and visceral organ size of low- and high-residual-feed-intake (RFI) broiler chickens fed either *ad libitum* or restrictively. Download Table S4, PDF file, 0.1 MB. Copyright © 2019 Metzler-Zebeli et al. 2019 Metzler-Zebeli et al. This content is distributed under the terms of the Creative Commons Attribution 4.0 International license.

10.1128/mSystems.00261-18.5 TABLE S5 Differences in intestinal histomorphology of low- and high-residual-feed-intake (RFI) broiler chickens fed either *ad libitum* or restrictively. Download Table S5, PDF file, 0.04 MB. Copyright © 2019 Metzler-Zebeli et al. 2019 Metzler-Zebeli et al. This content is distributed under the terms of the Creative Commons Attribution 4.0 International license.

In the mid-jejunum, restrictive feeding tended (*P = *0.086) to increase the level of expression of jejunal *MCT1* by 16.8% (Table S6). The *SMCT* gene, in turn, was differently expressed in the jejunum of high-RFI compared to low-RFI chickens, depending on the FL, as indicated by the FL × RFI interaction (*P = *0.035). With *ad libitum* feeding, the level of jejunal *SMCT* expression in low-RFI chickens was approximately one-third that in high-RFI chickens, whereas with restrictive feeding, the jejunal *SMCT* expression was 41.6% greater in low-RFI chickens than in high-RFI chickens. A similar trend (*P < *0.10) for a FL × RFI interaction with regard to the expression levels of *SMCT* and *SGLT1* was found for the cecal mucosa. Regardless of the FL, in contrast to the jejunal results, the levels of expression of cecal *MCT1* were higher (*P = *0.018) in both high-RFI chicken groups than in the low-RFI chicken group. With respect to the mucosal cytokine gene expression in the ceca, the expression levels of *NFKB* tended to be higher with low-RFI chickens than with high-RFI chickens as well as with restrictive feeding compared to *ad libitum* feeding (*P < *0.10), whereas the cecal expression of *TNFA* was higher only in restrictively fed high-RFI chickens and not in the other 3 groups (*P < *0.05).

10.1128/mSystems.00261-18.6 TABLE S6 Differences in expression levels of nutrient transporter, barrier, and innate immune genes at the jejunal mucosa of low- and high-residual-feed-intake (RFI) broiler chickens fed either *ad libitum* or restrictively. Download Table S6, PDF file, 0.1 MB. Copyright © 2019 Metzler-Zebeli et al. 2019 Metzler-Zebeli et al. This content is distributed under the terms of the Creative Commons Attribution 4.0 International license.

Electrophysiological data for the distal jejunum showed that the restrictive feeding decreased the short-circuit current (*I*_sc_) compared to *ad libitum* feeding, whereas the tissue resistance was higher in low compared to high-RFI chickens (*P < *0.05) (Table 3). When glucose was added mucosally, only the jejunal mucosa of *ad libitum*-fed chickens responded differently, as indicated by the FL × RFI interaction (*P < *0.05), with low-RFI chickens showing a 100% lower change in the tissue resistance than high-RFI chickens. Restrictively fed chickens, however, had similar tissue resistance responses to the added glucose.

**TABLE 3:** Differences in mucosal permeability and response to luminal glucose addition in distal jejunum of low- and high-RFI broiler chickens fed either *ad libitum* or restrictively^*a*^

Parameter	Value^*b*^				SEM	*P* value		
	*Ad libitum* feeding		Restrictive feeding					
	Low RFI	High RFI	Low RFI	High RFI		FL	RFI	FL × RFI
Avg electrophysiological variables								
*I*_sc_ (µA/cm^2^)	2.98	−0.29	−0.83	−0.97	1.029	0.036	0.107	0.137
*R_T_* (W/cm^2^)	0.66	0.58	0.69	0.60	0.034	0.495	0.022	0.912
FITC (nmol/cm^2^ × h)	0.0041	0.0055	0.0045	0.0032	0.0011	0.400	0.975	0.259
HRP (pmol/cm^2^ × h)	0.0094	0.0014	0.0019	0.0056	0.0038	0.654	0.577	0.130
								
Glucose response^*c*^								
Basal *I*_sc_ (µA/cm^2^)	6.53	5.66	4.74	4.43	0.817	0.073	0.480	0.733
Δ*I*_sc_	2.16	3.11	2.49	2.55	0.559	0.841	0.372	0.431
Basal *R_T_* (W/cm^2^)	0.58	0.54	0.59	0.57	0.031	0.628	0.270	0.837
Δ*R_T_*	3.65^*b*^	7.31^*a*^	5.12^*b*^	4.33^*b*^	1.048	0.475	0.180	0.041

a Data are presented as least-squares means and pooled SEM. *n *=* *7 per FL group, RFI rank, and sex except for *n *=* *8 high-residual-feed-intake (RFI) *ad libitum* females. RFI was calculated for the experimental period from 9 to 30 days posthatch. FL, feed intake level; FITC, fluorescein 5(6)-isothiocyanate; HRP, horseradish peroxidase.

b Different letters within a row indicate significant differences (*P *≤* *0.05).

c Data represent responses to glucose addition to reach a final chamber concentration of 5 mmol/liter. Δ*I*_sc_, difference between the maximal *I*_sc_ value obtained 2 min after glucose addition and the basal value determined 1 min before glucose addition; Δ*R_T_*, difference between the basal glucose transport (GT) value determined 1 min before glucose addition and the *R_T_* value obtained 2 min after glucose addition.

### FL- and RFI-related microbiota effects on the host.

**FIG 2:**
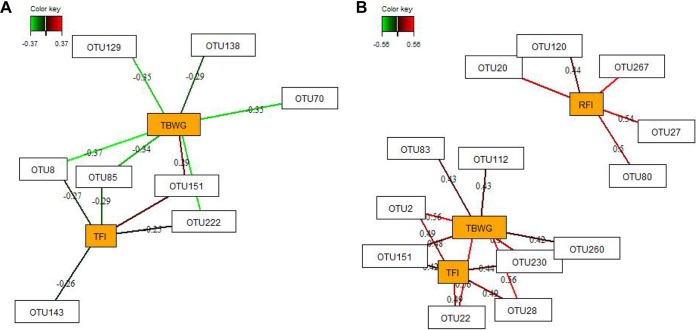
Determination of discriminant operational taxonomic units (OTUs) in ileal and cecal digesta for total feed intake (TFI), total body weight gain (TBWG), and residual feed intake (RFI) in low- and high-RFI chickens fed either *ad libitum* or restrictively. Covariations between the relative abundances of bacterial OTUs (relative abundances > 0.01) and TFI, TBWG, and RFI levels were assessed using sparse-partial-least-squares regression. The network is displayed graphically as nodes (OTUs and performance traits) and edges (biological relationship between nodes), with the edge color intensity indicating the level of the association as follows: red, positive; green, negative. Only the strongest pairwise associations were projected. Relevance networks for (A) relationships between OTUs in ileal digesta, TFI, and TBWG (*r *>* *0.25) and (B) relationships between OTUs in cecal digesta, RFI, TFI, and TBWG (*r *>* *0.40) are shown.

Relevance network analysis provided us with a predictive model to identify the most discriminant bacterial taxa (operational taxonomic units [OTUs]) in ileal and cecal digesta on chicken RFI and TFI and on performance. Thresholds were set for each network to present only the strongest pairwise associations as the most influential (Fig. 2). Ileal OTUs were discriminative for TFI and total body weight gain (TBWG), whereas only weak relationships with RFI were found that did not reach the threshold correlation level (|*r*| = 0.25). Only *Enterobacteriaceae* OTU151 was positively correlated with both TFI and TBWG. *Lactobacillus* OTU8, OTU85, and OTU222 were negatively correlated with TFI and TBWG, whereas *Ruminococcus* OTU143 was negatively associated only with TFI as well as *Lactobacillus* OTU70 and *Lactobacillus* OTU129 and *Turicibacter* OTU138 were negatively related only to TBWG (Fig. 2). In cecal digesta, 8 OTUs were found to be similarly discriminative for TFI and TBWG with the correlation threshold set to |*r*| = 0.40. These included several *Enterobacteriaceae* OTUs (OTU2, OTU22, OTU28, OTU151, and OTU230), *Clostridiales* OTU112, *Turicibacter* OTU83, and *Blautia* OTU260. In the ceca, 5 OTUs (3 *Anaerotruncus* OTUs [OTU20, OTU27, and OTU267], *Ruminococcus* OTU80, and *Clostridiales* OTU120) could be distinguished that were influential with respect to the RFI.

**FIG 3:**
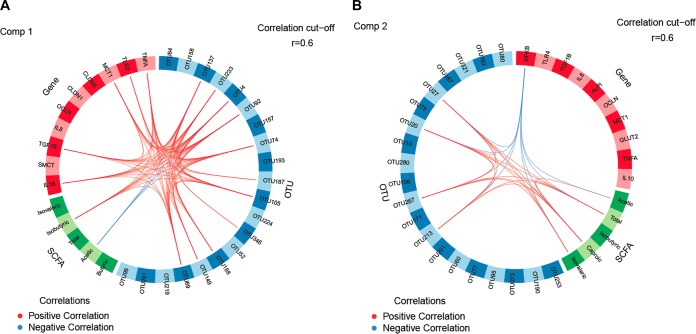
Circos plots of horizontal sparse partial least-squares-discriminant analysis displaying correlations between the identified levels of the best discriminant operational taxonomic units (OTUs; *n *=* *20) and short-chain fatty acids (SCFA, *n *=* *5) and expression levels of target genes (*n *=* *10) for (A) component 1 and (B) component 2 in the cecum. Positive and negative correlations (*r *>* *0.6) are displayed by red and blue links, respectively. Relative abundance of bacterial OTUs = >0.01%.

Relationships between the bacterial presence and the host response in the ceca were further investigated with multigroup supervised sparse partial least-squares-discriminant analysis (sPLS-DA) (13) to identify OTUs, genes, and SCFAs that contributed best to the discrimination of component 1 and component 2 data between FL and RFI groups (Fig. 3). The sPLS-DA identified 14 OTUs within the unclassified *Clostridiales* family in the cecal digesta as most discriminant for the first component (Fig. 3A), some of which were positively associated with the host mucosal expression of *TNFA*, *TLR2*, *MCT1*, *TGFB1*, and *IL1B* and with the cecal isobutyrate concentration. By contrast, the acetate concentration was negatively correlated with 4 *Clostridiales* OTUs (OTU4, OTU74, OTU92, and OTU137). For component 2, the expression level of *NFKB* in the cecal mucosa was negatively associated with 3 *Anaerotruncus* OTUs (OTU20, OTU27, and OTU267) and *Clostridiales* OTU213 as well as with the concentrations of total SCFA, acetate, isovalerate, and caproate (Fig. 3B). The same *Anaerotruncus* and *Clostridiales* OTUs appeared to enhance total SCFA isovalerate and caproate levels in cecal digesta for component 2.

## DISCUSSION

In the present study, we used restrictive feeding to impose the same feed intake on all chickens in order to clarify whether the RFI-associated variation in chicken’s feed intake is a factor vital to RFI-related differences in intestinal microbial composition, physiology, and function. In doing so, we observed a strong impact of restrictive feeding on the intestinal physiology and bacterial communities, mainly influencing the predominant families, including *Turicibacteraceae*, *Ruminococcaceae*, and *Enterobacteriaceae* in ileal and cecal digesta. Relevance network analysis further supported the idea that, in particular, the abundance of *Lactobacillus* (negative) in ileal digesta, *Turicibacter* (positive) in cecal digesta, and *Enterobacteriaceae* (positive) in both intestinal segments depended on chicken’s feed intake. By contrast, only a few RFI-associated taxa were detectable; in particular, *Anaerotruncus* OTUs were discriminative for high RFI. Overall, more RFI-associated differences were found for the host physiology, i.e., shallower crypts and fewer goblet cells in ceca and a trend toward a lower-weight liver in low-RFI chickens, which may be explained by host-related energy-saving mechanisms associated with low RFI (5). In contrast to our hypothesis, the restrictively fed high-RFI chickens often showed intestinal profiles (e.g., SCFA profile and size of the pancreas) that differed from those shown by the other three chicken groups as indicated by the FL × RFI interactions. These findings suggest the presence of host-related physiological adaptations, presumably in relation to the RFI-associated greater energy and nutrient needs of high-RFI chickens, to compensate for the restricted feeding and hence reduced intestinal nutrient availability and may help to explain the improved RFI values in the restrictively fed high-RFI birds described here.

By restriction of the total amount of feed available, chickens received lower levels of energy sources as well as of macro- and micronutrients than their *ad libitum*-fed counterparts. Because low-RFI chickens typically eat less feed than high-RFI chickens (12), the feed restriction was more severe for the high-RFI chickens than for the low-RFI chickens. The two characteristic physiological adaptations in the restrictively fed chickens were a bigger crop and a bigger duodenum, possibly to enhance the utilization of the feed, irrespective of the RFI rank. Further in-depth experimentation is required to determine whether the enlargement of the crop was related to increased retention time or to bacterial activity with respect to the feed in this segment. Intriguingly, only the restrictively fed high-RFI chickens had a larger pancreas and not their low RFI counterparts, which may explain the greater nutrient retention in these birds than in the birds in the other 3 groups (4). This finding may imply higher energy and nutrient needs for basal metabolism in high-RFI chickens than in low-RFI chickens (1) which would be met by the increased feed intake in high-RFI chickens under *ad libitum* feeding conditions (12). We did not perform histomorphological measurements for the duodenum to evaluate changes in the villus surface as we mainly focused on the jejunum and ileum. There, the absorptive and secretory surfaces were similar among the chicken groups.

The lower (negative) jejunal *I*_sc_ in restrictively fed chickens indicated a greater net absorption of anions (i.e., chloride and bicarbonate) than of cations compared to *ad libitum*-fed chickens (14). The similar *I*_sc_ responses seen after stimulation of active Na^+^-coupled glucose transport (SGLT1) via mucosal glucose addition in the Ussing chambers and similar expression levels of *SGLT1* and *GLUT2* among chicken groups indicated, however, that neither restrictive feeding nor RFI rank influenced chicken’s jejunal capacity to actively absorb glucose. By contrast, high-RFI chickens appeared to take up more glucose in a paracellular manner as indicated by the FL × RFI interaction for the delta transepithelial resistance (*R_T_*). As this effect was not detected with restrictive feeding, it is likely that the RFI-associated differences in the feed intake of the chickens represented the driving force behind this RFI-related physiological adaptation. Concurrently, low-RFI chickens had a higher barrier function as indicated by the higher levels of jejunal tissue resistance than were seen with high-RFI chickens, suggesting RFI-related differences in host metabolism and paracellular nutrient absorption, as the effects were similar for the two feeding-level groups. Restrictive feeding, however, did not impair the mucosal barrier function as previously reported (11), as shown by equal jejunal permeability marker flux rates and *R_T_* values and tight-junction protein expression levels.

It is likely that, as a consequence of enhanced nutrient uptake in the upper small intestine, fewer nutrients reached the ileum and ceca of restrictively fed high-RFI chickens, thereby leading to modulation of the bacterial composition and activity in those segments. It can thus be speculated that the decrease in ileal acetate levels and hence in total SCFA concentrations may have been a consequence of the drastic decline in *Turicibacteraceae* and *Enterobacteriaceae* levels in restrictively fed chickens. However, since the proportional contributions of the single SCFA hardly changed, the lower substrate availability in restrictively fed birds than in *ad libitum*-fed birds may have been the main factor responsible for the lower SCFA concentration in ileal digesta. Concurrently, the FL × RFI interactions for SCFA may have supported the lower ileal substrate flow in the restrictively fed high-RFI chickens than in the chickens in the other 3 groups, which apparently promoted the metabolic activity of propionate-, valerate-, caproate-, and isovalerate-producing species. Overall, the ileal isobutyrate levels contributed considerably to the ileal SCFA concentrations across all chicken groups and may have been related to the high protein content of the starter, grower, and finisher diets, as branched-chain fatty acids mainly result from fermentation of branched-chain amino acids (15). Although SCFA levels were not measured in jejunal digesta, the FL × RFI interactions for the jejunal and cecal *SMCT* expression levels may indicate that changes in SCFA profiles underlie these findings. For instance, the higher proportion of cecal butyrate in both the *ad libitum*-fed high-RFI and restrictively fed low-RFI chicken groups showed the same pattern as the trend toward *SMCT* expression levels in these 2 chicken groups compared to the other 2 groups. Since butyrate is one of the primary energy sources for the intestinal mucosa (16), an enhanced rate of mucosal butyrate uptake may be behind the trend toward an enlargement of the ceca among the restrictively fed low-RFI chickens compared to the other 3 groups.

With respect to the bacterial taxonomy, however, FL × RFI interactions were absent, indicating that the intestinal substrate availability may have more extensively affected the metabolic capacities of bacteria than the actual abundances. In support of this idea, both restrictively fed chicken groups had higher species richness and evenness in their ileal digesta than the *ad libitum*-fed chickens, suggesting a robust effect of restrictive feeding on the taxonomic richness of the bacterial microbiome. By contrast, RFI-associated bacterial differences were more or less absent in the ileum as indicated by diversity data and taxonomic and networking analyses. This may have been related to the fact that, although the *ad libitum*-fed high-RFI chickens ate more than the low-RFI chickens, the nutrient profiles in digesta of *ad libitum*-fed low- and high-RFI chickens are similar, as can be assumed from the equal levels of nutrient retention in these birds (4). Following this concept, the restrictively fed low-RFI chickens should have had a bacterial profile similar to that shown by the *ad libitum*-fed birds, which was not true in the present study. Therefore, aside from the total amount of nutrients available, other physiological adaptations caused by restrictive feeding may explain the present restrictive-feeding-associated taxonomic differences. Possible changes in the small intestinal passage (7), together with the enlarged crop, may have given the microbiota more time to utilize nondigested dietary material. Accordingly, *Lactobacillaceae*, *Ruminococcaceae*, and *Lachnospiraceae,* which have functional abilities to degrade nondigestible carbohydrates (e.g., resistant starch, hemicellulose, and cellulose) (17, 18), were enriched with restrictive feeding in the ileum. *Lactobacillaceae*, *Ruminococcaceae*, and *Lachnospiraceae* are able to bind to and degrade host glycoproteins (19–21). Therefore, a greater mucus secretion to facilitate the digesta flow in restrictively fed chickens might have been another underlying mechanism. However, jejunal and ileal goblet cell numbers as well as the jejunal expression levels of *MUC1* and *MUC2* did not indicate changes in levels of mucus production between the chicken groups. In line with this, the decrease in the level of mucin-degrading *Enterobacteriaceae* (22) seen with restrictive feeding also indicates that dietary substrate-related changes are likely the cause for alterations in the ileal community.

The NMDS-based β-diversity analysis supported the idea of the importance of the intestinal nutrient flow for the bacterial communities in the ceca. In fact, the cecal microbiota of the *ad libitum*-fed high-RFI chicken group, which was the chicken group with the highest nutrient flow into the ceca, clustered distinctly from the cecal community of restrictively fed high-RFI chickens, with presumably the lowest cecal flow among the four chicken groups. Moreover, a depletion of the cecal digesta in their preferred substrate may have caused the restrictive-feeding-associated reduced abundances of *Ruminococcaceae* and *Lachnospiraceae*, which comprise important acetate- and butyrate-producing species (17, 23, 24), thereby providing a growth advantage for other *Clostridiales* bacteria. According to the changes in SCFA profiles, the latter may have comprised several propionate producers and protein utilizers. These assumptions are supported by the integration analysis data, showing positive and negative correlations for isobutyrate and acetate levels, respectively, with several unclassified *Clostridiales* species (e.g., OTU4, OTU74, OTU92, and OTU137) for component 1. Despite the limitations of predictions of metabolic capabilities from unclassified OTUs, circos plots indicated that these OTUs may have modified the cecal mucosal response related to *TLR2* and cytokine signaling (e.g., *TNFA*) as well as SCFA uptake.

Reduced energy and nutrient availability in restrictively fed chickens may have impaired the adaptive mucosal immune defense in the ileum and ceca as indicated by the trend toward lower levels of lymphocytes in the lamina propria in those chickens than in *ad libitum*-fed chickens. Notably, cecal *NFKB* expression levels were lower in high-RFI chickens than in low-RFI chickens. According to the circos plot for component 2, this may have been related to the inhibitory effect of cecal SCFA (i.e., acetate, isovalerate, and caproate) on proinflammatory signaling pathways (16), which, in turn, seemed to be related to the increased *Anaerotruncus* abundance in cecal digesta of high-RFI chickens compared to low-RFI chickens.

In conclusion, the present results provided novel information with respect to the importance of the FL for RFI-associated intestinal bacterial and host physiological variation in chickens. By using quantitative restrictive feeding to impose the same feed intake on all chickens, we were able to show that restrictive feeding was the main driver for the ileal and cecal abundances of the predominant bacterial families *Turicibacteraceae*, *Ruminococcaceae*, and *Enterobacteriaceae*. In contrast, the cecal abundance of *Anaerotruncus* was mainly associated with high RFI, a finding supported by relevance network analysis. Results also demonstrated host-related physiological adaptations in response to the RFI-associated nutritional needs of high-RFI chickens to compensate for the restricted feeding and reduced intestinal nutrient availability. This may have contributed to the decreased RFI level in restrictively fed high-RFI chickens. In addition, low-RFI chickens developed energy-saving mechanisms (i.e., shallower crypts and fewer goblet cells in ceca and a trend toward a lower-weight liver) and a stronger jejunal barrier function. Data corresponding to dependencies among each bird’s cecal microbiota and SCFA levels and expression levels of innate immune and SCFA transporter genes were further supported by multigroup supervised integration analysis.

## MATERIALS AND METHODS

### Ethical approval.

All experimental procedures, including animal handling and treatment, were approved by the institutional ethics committee of the University of Veterinary Medicine Vienna and the Austrian national authority according to paragraph 26 of the Law for Animal Experiments, Tierversuchsgesetz 2012—TVG 2012 (GZ 68.205/0148-II/3b/2015).

### Animals, housing, and experimental design.

One-day-old Cobb 500 female (*n *=* *57) and male (*n *=* *55) broiler chicks were used in 2 consecutive replicate batches (56 chickens per batch), with 1 more female and 1 fewer male in batch 1 than in batch 2. Housing and environmental conditions have been previously described (12). All chickens had free access to demineralized water from manual drinkers and were fed the same starter (1 to 8 dph), grower (9 to 20 dph), and finisher (21 to 37 dph) corn-soybean meal-based diets (see Table S7 in the supplemental material). All diets were free of antibiotics and coccidiostatics. From 1 to 8 dph, chicks (*n *=* *5 to 6) of the same sex were housed in groups in stainless steel metabolic cages and had *ad libitum* access to feed to ensure sufficient feed intake in the first days of life. From 9 dph until the end of the experiment (33 to 37 dph), chickens were individually housed to determine their individual levels of feed intake and were randomly assigned to 2 different treatments. Half of the chickens had *ad libitum* access to feed (for both replicate batches together, *n *=* *29 females and *n *=* *28 males), whereas the other half were restrictively fed (for both replicate batches together, *n *=* *28 females and *n *=* *27 males). The daily feed allocation of restrictively fed chickens was aimed to correspond to 90% to 95% of the average daily *ad libitum* feed intake observed in a former chicken trial in which we assessed the FE of the same chicken line (12). Optimally, feeders of the restrictively fed chickens were empty the next morning. A further aim was that all chickens in the restrictive feeding group would eat the same amount of feed. Therefore, the feed amount for the restrictively fed chickens was additionally adjusted daily based on observations of the female and male chickens with the lowest feed intake the day before. For both treatment groups, fresh feed was provided at 9:00 h, and feeders were refilled at 15:00 h.

10.1128/mSystems.00261-18.7 TABLE S7 Dietary ingredients and chemical composition of diets (on as-fed basis). Download Table S7, PDF file, 0.03 MB. Copyright © 2019 Metzler-Zebeli et al. 2019 Metzler-Zebeli et al. This content is distributed under the terms of the Creative Commons Attribution 4.0 International license.

### Determination of FE.

For both treatment groups, the feed intake of each chicken was recorded on 9, 14, 21, 28, and 30 dph. In order to determine the feed intake, feed refusals were collected daily before morning feeding, and feed spills were collected weekly. Chickens were weighed on dph 1, 7, 9, 14, 21, 28, and 30. The RFI used as a metric for FE was determined for the period from 9 to 30 dph. For this, TFI, metabolic mid-test BW (MMW), and TBWG were used to estimate each chicken’s RFI as the residuals over the test period (9 and 30 dph) using a nonlinear mixed model (SAS Stat Inc., version 9.4; Cary, NC, USA) (12). In each replicate batch (separately for females and males and balanced for batch), the chickens with the lowest RFI (good FE) and highest RFI (poor FE) values in each FL group were selected to be sampled. This resulted in 14 low-RFI (*n *=* *7 per sex) and 15 high-RFI (*n *=* *8 females; and *n *=* *7 males) *ad libitum*-fed chickens and 14 low-RFI (*n *=* *7 per sex) and 14 high-RFI (*n *=* *7 per sex) restrictively fed chickens in both batches together. Intestinal parameters were analyzed for only those selected chickens.

### Sample collection.

Chickens were weighed before being euthanized with an overdose of thiopental (medicamentum pharma GmbH; Allerheiligen im Mürztal, Austria) (50 to 100 mg/kg of body weight) by intravenous (i.v.) injection into the caudal tibial vein between 33 and 37 dph. After the abdominal cavity was opened, the visceral organs were removed and their weight was recorded using a method similar to that described previously by Metzler-Zebeli et al. (5). Visceral organ weight and total and segmental intestinal length were expressed per kilogram of body weight to account for differences in body weight among individual chickens. Intestinal digesta was collected and thoroughly homogenized using a spatula before snap-freezing in liquid nitrogen and long-term storage at −80°C for microbiota analysis or short-term storage on ice and storage at −20°C for SCFA analysis. Pieces of the intestinal tube (1-cm diameter) for morphometric measurements were collected from the Meckel’s diverticulum, the first centimeter of the proximal ileum, and proximal to the blind end of the ceca. Those pieces were thoroughly washed in phosphate-buffered saline and fixed in neutrally buffered (pH 7.0) formalin (4% [vol/vol]). A 20-cm-long tissue tube piece for the Ussing chamber experiment was collected distal to the Meckel’s diverticulum, immediately transferred into ice-cold and pregassed (carbogen gas [95% O_2_–5% CO_2_]) transport buffer (5), and transported to the laboratory within 10 min of the death of the animal. Thereafter, the remaining intestinal segments were opened at the mesenterium, washed in neutrally buffered saline, and blotted dry with paper tissue. A glass slide was used to scrape the mucosa from the jejunum between the Meckel’s diverticulum and 35 cm toward the duodenum as well as from both ceca. Mucosa samples were immediately snap-frozen in liquid nitrogen, and aliquots were stored at −80°C for RNA isolation.

### Histomorphology.

Histomorphological measurements were performed as described previously by Metzler-Zebeli et al. (5). After fixation of the intestinal tube pieces, the tube pieces were dehydrated in ethanol, cleared in xylene, and embedded in paraffin. Three discontinuous 3-to-4-µm-thick sections per intestinal site were routinely stained with hematoxylin and eosin and examined on a Leica DM2000 light microscope (Leica Microsystems, Wetzlar, Germany) fitted with a digital camera (Leica DFC425C) and using Leica Application Suite V3.7 software. The images were analyzed with ImageJ software (Version 1.47; National Institutes of Health, MD, USA). In total, 15 intact well-oriented, crypt-villus units were selected, with the criteria for villus selection based on the presence of intact lamina propria. Villus height and width were measured at ×4 magnification and crypt depth at ×10 magnification. The circular and longitudinal muscular layers were measured. Numbers of goblet cells per 250 μm of villus or crypt epithelium were determined using 15 replicates per intestinal section at ×10 magnification. Intraepithelial lymphocytes were counted per 400 µm of villus epithelium using 12 replicates per intestinal section at ×20 magnification.

### Intestinal electrophysiology.

Differences in intestinal electrophysiological parameters and permeability marker flux were evaluated for four chickens per sampling day (5, 25). This resulted in six observations per RFI and FL group and sex. The jejunal tube pieces were opened at the mesenterium and rinsed with transport buffer. Clean tissue pieces were stripped of the outer serosal layers (5). Three consecutive pieces were cut from the proximal 10 cm of the jejunal tube, mounted in Ussing chambers (exposed area of 0.91 cm^2^), and incubated in a total volume of 10 ml of serosal and mucosal buffer solution (pH 7.4, 38°C) (5). Continuous gassing with carbogen using a gas lift was provided on both the mucosal and the serosal sides to ensure oxygenation and circulation of the buffer. In using two pairs of dual-channel current and voltage Ag-AgCl electrodes, which were connected via 3% agar bridges filled with 3 M potassium chloride, the potential difference (mV), *I*_sc_ (measured in microamperes per square centimeter), and *R_T_* (Ω × square centimeter) were continuously recorded using a microprocessor-based voltage-clamp device and software (version 9.10) (Microclamp; Mussler, Aachen, Germany). The tissue was alternatively pulsed with a positive or negative pulse of 20 µA and 100-ms duration. After an equilibration period of 20 min under open-circuit conditions, the tissue was short-circuited by clamping the voltage to zero. After electrophysiological measurements were recorded for 5 min, fluorescein 5(6)-isothiocyanate (FITC) (Sigma-Aldrich, Schnelldorf, Austria) (389.38 g/mol) and horseradish peroxidase (HRP) (Carl Roth GmbH & Co. KG, Karlsruhe, Germany) (44,000 g/mol) were added to reach final concentrations of 0.1 mM and 1.8 µM, respectively, to assess the mucosa-to-serosa flux (5). The glucose-absorptive activity of the tissue samples was assessed by adding glucose to reach a final concentration of 10 mmol/liter to the buffer at the mucosal side at 45 min after short-circuiting the tissue (25). The chemical effect on glucose transporter function was measured by comparing the *I*_sc_ and *R_T_* values for 1 min before glucose was added to the peak current, and the resistance response of the exposed tissue (Δ*I*_sc_ and Δ*R_T_*) was obtained within 2 min after the addition of glucose.

### Mucosal gene expression.

Total RNA was isolated from jejunal mucosal scrapings of low- and high-RFI chickens (26) using mechanical homogenization (FastPrep-24 instrument; MP Biomedicals, Santa Ana, CA) and the RNeasy Mini kit (Qiagen, Hilden, Germany). The RNA isolates were treated with DNase I (RNA Clean & Concentrator-5 kit, Zymo Research, Irvine, USA) before transcribing 2 µg of total RNA into single stranded cDNA using the High Capacity Reverse Transcription kit (Life Technologies Foster City, USA). The quality (q) of the isolated RNA was verified using the Agilent 2100 Bioanalyzer (Agilent Technologies, Santa Clara, USA), showing RNA integrity numbers between 8 and 10.

Primers for target genes and potential housekeeping genes (HKG) used were published previously by our group (5) (Table S8). Amplifications were performed on the Stratagene Mx3000P QPCR System (Agilent Technologies, Santa Clara, CA) using the following conditions: 95°C for 5 min, followed by 95°C for 10 s, 60°C for 30 s, and 72°C for 30 s for 40 cycles, followed by the generation of dissociation curves. Each 20 µl reaction consisted of 50 ng cDNA, 10 µl Fast Plus Eva Green master mix with low ROX (Biotium, Hayward, CA, USA), 100 nM (each) forward and reverse primers, and DEPC-treated water in a 96-well plate (VWR, Vienna, Austria). All reactions were run in duplicate. Negative controls and reverse transcription controls (*R_T_* minus) were included in order to control for residual DNA contamination. Of the 6 tested HKG, *ACTB* and *B2M* were the most stably expressed, and their data were analyzed using NormFinder (27) and BestKeeper (28). The geometric mean of the expression levels of *ACTB* and *B2M* was used for normalization of target gene expression levels. For this, the mean raw gene expression data (obtained as quantification cycle [*Cq*] values) from the identified HKG were subtracted from the *Cq* values determined for the target genes to determine Δ*Cq* values. Gene expression levels were calculated relative to those determined for the chicken with the lowest expression of the corresponding genes using the 2^−ΔΔ^*^Cq^* method. Data representing the amplification efficiencies [E = 10^(−1/slope)−1^] of all primer sets are provided in Table S2 and were prepared by using a 5-fold serial dilution of samples.

10.1128/mSystems.00261-18.8 TABLE S8 Oligonucleotide nucleotide primers used for the gene expression experiment. Download Table S8, PDF file, 0.04 MB. Copyright © 2019 Metzler-Zebeli et al. 2019 Metzler-Zebeli et al. This content is distributed under the terms of the Creative Commons Attribution 4.0 International license.

### Short-chain fatty acid analysis.

Individual SCFA concentrations in ileal and cecal digesta were determined using gas chromatography (29). Briefly, 1 g of ileal or cecal digesta was mixed with 0.2 ml of 25% metaphosphoric acid, 1 ml of double-distilled water, and 200 µl of internal standard (4-methyl-valeric acid; Sigma-Aldrich, Vienna, Austria) and was centrifuged at 3,148 × *g* for 10 min (5810 R centrifuge; Eppendorf, Hamburg, Germany). The supernatant was centrifuged at 15,000 × *g* for 25 min (5424 centrifuge; Eppendorf), and the clear supernatant was analyzed for SCFA (acetate, propionate, butyrate, isobutyrate, valerate, isovalerate, and caproate) using gas chromatography.

### DNA extraction and 16S rRNA gene sequencing.

Total DNA was isolated from 250 mg of ileal samples (*n *=* *57) and cecal samples (*n *=* *57) using a PowerSoil DNA isolation kit (MoBio Laboratories Inc., Carlsbad, CA, USA) with modifications performed as previously described (30). The DNA concentration was quantified using a Qubit 2.0 Fluorometer (Life Technologies, Carlsbad, CA, USA) and a Qubit double-stranded DNA (dsDNA) HS assay kit (Life Technologies). An aliquot of each of the DNA samples was sent to a commercial provider (Microsynth AG, Balgach, Switzerland). The V3-V5 hypervariable region of the 16S rRNA gene was amplified using primers 357F-HMP (5′-CCTACGGGAGGCAGCAG-3′) and 926R-HMP (5′-CGTCAATTCMTTTRAGT-3′) to generate an approximate amplicon size of 570 bp (31) and a Kapa HiFi HotStart PCR kit (Roche, Baden, Switzerland), which included high-fidelity DNA polymerase. Libraries were constructed by ligating sequencing adapters and indices onto purified PCR products using a Nextera XT sample preparation kit (Illumina Inc., San Diego, CA, USA) and the recommendations of the manufacturer. Equimolar amounts for each library were pooled and sequenced on an Illumina MiSeq Personal Sequencer using a 300-bp read length paired-end protocol. Afterwards, FASTQ files were demultiplexed, quality filtered, and trimmed of Illumina adaptor residuals, and the overlapping paired-end reads were stitched by Microsynth.

### Bioinformatic analysis.

Sequence data were analyzed with Quantitative Insights Into Microbial Ecology (QIIME) package version 1.9.1 (32). After quality trimming of the stitched reads using a quality threshold of q = >20, the UCHIME method (using the 64-bit version of USEARCH [33, 34]) and the GOLD database were used to screen for and exclude chimeric sequences. Open-reference OTU picking was done at the 97% similarity level using UCLUST (Edgar [33]) and the Greengenes database (version 13_8) as the reference template (35). Rare OTUs with fewer than 10 sequences were removed. For α-diversity (Shannon, Simpson, and observed species) analyses, samples were rarefied to a depth of 2,079 sequences.

### Statistical analyses.

To identify the most influential OTUs and SCFAs in cecal digesta and cecal mucosal expression of genes and SCFAs with regard to the FL and each chicken’s RFI, multigroup supervised DIABLO N-integration networking was performed by means of the package “mixOmics” (version 6.3.2) (13) in R studio (version 1.0.136). Horizontal sparse partial least-squares-discriminant analysis (sPLS-DA) was used to integrate the data sets of relative abundances of OTUs, SCFAs, and mucosal expression levels of target genes in order to classify and select key features from each data set. Tuning of sPLS-DA parameters was performed to determine the main OTUs and SCFAs and the mucosal expression levels of genes that enable discrimination of treatments groups with the lowest possible error rate, resulting in selection of 20 OTUs and 5 SCFAs and 10 genes each for components 1 and 2, respectively. The sPLS-DA results were visualized as circos plots showing the strongest positive and negative Pearson’s correlations (|*r*| > 0.6) between the most discriminant OTUs and SCFAs and the mucosal expression levels of genes for each subset of data and identified features. Additionally, sPLS and relevance network analyses were performed using mixOmics (13, 36) to integrate data of OTUs (0.01% of all reads) in ileal and cecal digesta with the results determined for TFI, TBWG, and RFI. Relevance network graphs from sPLS were obtained via the function network. For β-diversity analysis, statistical assessment of dissimilarity (Bray-Curtis) matrices derived from OTU data was performed with PERMANOVA using the “adonis2” function and visualized in two-dimensional nonmetric multidimensional scaling (NMDS) ordination plots obtained with the “metaMDS” function in the vegan R package (version 2.5.2) (37).

After analyzing for normality using the Shapiro-Wilk test (version 9.4; SAS Stat Inc., Cary, NC, USA), FE parameters, data of intestinal microbiota at different taxonomic levels and of SCFA as well as data of intestinal size, structure, and function were subjected to ANOVA using the MIXED procedure in SAS. With regard to the microbiome data, only the bacterial phyla and families comprising a relative abundance level of >0.01% of all reads across both sexes as well as the 40 most discriminative OTUs identified in the sPLS-DA were subjected to ANOVA. The fixed effects of batch, sex, FL, and RFI and of the two-way-interaction FL × RFI were considered in the main model. The batch effect was considered a random effect in the final model. Chicken data nested within batch data represented the experimental unit. Degrees of freedom were approximated using the Kenward-Roger method. Differences among least-squares means were computed using the pdiff statement. Differences were considered significant for *P* values of *≤*0.05 and were considered to represent trends for *P* values greater than 0.05 and less than or equal to 0.10. Sex was significant for the FE and performance data; therefore, a second model for these parameters was adjusted and data were additionally analyzed separately for females and males.

### Data availability.

Raw sequencing data are available in NCBI’s BioProject SRA database (accession no. PRJNA495920).
