# Supplementary material for: Feed Restriction Modifies Intestinal Microbiota-Host Mucosal Networking in Chickens Divergent in Residual Feed Intake
Source: mSystems. 2019 Jan 29;4(1):e00261-18. doi: 10.1128/mSystems.00261-18 (PMC6351724; doi:10.1128/mSystems.00261-18)
Supplement: TABLE S1 [file mSystems.00261-18-st001.pdf]

TABLE S1 Total feed intake (TFI), total body weight gain (TBWG), and residual feed intake (RFI) values of low and high RFI broiler chickens fed either *ad libitum* or restrictively[1-3]

	Ad libitum		Restrictive			*P* value		
Item	Low RFI	High RFI	Low RFI	High RFI	SEM	FL[4]	RFI	FL × RFI
Both sexes								
TFI, g	2337[b]	2620[a]	2110[c]	2171[c]	52.5	<0.001	0.002	0.040
TBWG, g	1696	1684	1501	1416	41.1	<0.001	0.242	0.376
RFI, g	-81[c]	226[a]	-67[c]	111[b]	23.0	0.033	<0.001	0.007
Females								
TFI, g	2146[b]	2578[a]	1965[c]	2074[bc]	57.7	<0.001	<0.001	0.010
TBWG, g	1529	1610	1354	1328	47.9	<0.001	0.570	0.279
RFI, g	-56[c]	271[a]	-34[c]	118[b]	35.7	0.077	<0.001	0.021
Males								
TFI, g	2529	2652	2255	2269	84.9	0.001	0.428	0.525
TBWG, g	1862	1753	1649	1503	65.9	0.002	0.066	0.784
RFI, g	-107	179	-99	104	29.0	0.256	<0.001	0.171

[1]Data are presented as least-square means and pooled SEM. *n* = 7 per FL group, RFI rank, and sex; except for *n* = 8 high RFI *ad libitum* females.

[2]TFI, TBWG, and RFI were calculated for the experimental period from 9 to 30 days post-hatch.

[3]Sex affected TFI, TBWG ($P \leq 0.001$), and RFI ($P \leq 0.05$).

[4]FL, feed intake level.

[a,b,c]Different superscripts within a row indicate significant difference ($P \leq 0.05$).
