# Supplementary material for: Feed Restriction Modifies Intestinal Microbiota-Host Mucosal Networking in Chickens Divergent in Residual Feed Intake
Source: mSystems. 2019 Jan 29;4(1):e00261-18. doi: 10.1128/mSystems.00261-18 (PMC6351724; doi:10.1128/mSystems.00261-18)
Supplement: TABLE S2 [file mSystems.00261-18-st002.pdf]

TABLE S2 Alpha-diversity of bacterial microbiota communities in ileal and cecal digesta of low and high residual feed intake (RFI) broiler chickens fed either *ad libitum* or restrictively[1,2]

	Ad libitum feeding		Restrictive feeding			*P* value		
Parameter	low RFI	high RFI	low RFI	high RFI	SEM	FL[3]	RFI	FL × RFI
Ileum								
Observed species	50	52	84	91	15.882	0.026	0.794	0.870
Shannon	2.19	2.20	3.00	3.22	0.288	0.002	0.678	0.718
Simpson	0.59	0.60	0.70	0.76	0.047	0.007	0.420	0.659
Ceca								
Observed species	286	274	303	297	10.258	0.060	0.406	0.785
Shannon	5.71	5.67	5.85	5.73	0.123	0.394	0.518	0.747
Simpson	0.94	0.94	0.95	0.94	0.008	0.643	0.630	0.558

[1]Data are presented as least-square means and pooled SEM. *n* = 7 per FL group, RFI rank, and sex; except for *n* = 8 high RFI *ad libitum* females.

[2]RFI was calculated for the experimental period from 9 to 30 days post-hatch.

[3]FL, feed intake level.
