# Supplementary material for: Feed Restriction Modifies Intestinal Microbiota-Host Mucosal Networking in Chickens Divergent in Residual Feed Intake
Source: mSystems. 2019 Jan 29;4(1):e00261-18. doi: 10.1128/mSystems.00261-18 (PMC6351724; doi:10.1128/mSystems.00261-18)
Supplement: TABLE S3 [file mSystems.00261-18-st003.pdf]

TABLE S3 Differences in relative abundance (%) of the dominant bacterial phyla present in ileal and cecal digesta of low and high residual feed intake (RFI) broiler chickens fed either *ad libitum* or restrictively[1,2]

	Ad libitum feeding		Restrictive feeding			*P* value		
Phylum	low RFI	high RFI	low RFI	high RFI	SEM	FL[3]	RFI	FL × RFI
Ileum								
Firmicutes	62.3	77.0	81.9	88.4	7.130	0.034	0.145	0.570
Proteobacteria	37.5	22.9	17.8	11.3	7.152	0.033	0.146	0.574
Tenericutes	0.023	0.0078	0.061	0.071	0.033	0.134	0.949	0.700
Cyanobacteria	0.13	0.10	0.23	0.18	0.086	0.289	0.665	0.912
Actinobacteria	0.0049	0.012	0.013	0.089	0.037	0.253	0.269	0.357
Ceca								
Firmicutes	91.3	92.2	96.4	98.2	1.548	<0.001	0.383	0.772
Proteobacteria	7.82	6.62	2.34	0.90	1.571	<0.001	0.405	0.939
Tenericutes	0.61	0.90	1.00	0.69	0.190	0.648	0.975	0.117
Cyanobacteria	0	0.00003	0	0.0008	0.00038	0.339	0.308	0.339
Actinobacteria	0.114	0.100	0.090	0.060	0.019	0.106	0.262	0.693

[1]Data are presented as least-square means and pooled SEM. *n* = 7 per FL group, RFI rank, and sex; except for *n* = 8 high RFI *ad libitum* females.

[2]RFI was calculated for the experimental period from 9 to 30 days post-hatch.

[3]FL, feed intake level.
