# Supplementary material for: Feed Restriction Modifies Intestinal Microbiota-Host Mucosal Networking in Chickens Divergent in Residual Feed Intake
Source: mSystems. 2019 Jan 29;4(1):e00261-18. doi: 10.1128/mSystems.00261-18 (PMC6351724; doi:10.1128/mSystems.00261-18)
Supplement: TABLE S4 [file mSystems.00261-18-st004.pdf]

TABLE S4 Differences in body weight at sampling and visceral organ size of low and high residual feed intake (RFI) broiler chickens fed either *ad libitum* or restrictively[1,2]

	Ad libitum feeding		Restrictive feeding			*P* value		
Parameter	low RFI	high RFI	low RFI	high RFI	SEM	FL[3]	RFI	FL × RFI
Body weight at sampling (kg)	2.43	2.46	2.40	2.31	0.07	0.234	0.770	0.416
Empty weight (g/kg BW)								
Crop	3.7	3.5	4.6	5.0	0.38	0.002	0.833	0.421
Proventriculus	4.2	4.0	4.0	4.1	0.25	0.714	0.993	0.503
Gizzard	14.0	13.3	13.7	14.3	0.74	0.655	0.953	0.351
Duodenum	5.5	5.5	5.9	6.3	0.22	0.011	0.342	0.321
Jejunum	16.0	16.7	16.6	16.8	0.93	0.700	0.638	0.733
Ileum	2.1	2.3	2.4	2.4	0.12	0.107	0.297	0.611
Cecum (average of the two)	1.1	1.1	1.3	1.1	0.06	0.074	0.084	0.105
Colon	1.4	1.3	1.4	1.5	0.08	0.409	0.758	0.220
Total intestine	76.8	75.5	79.2	81.9	2.67	0.107	0.796	0.469
Pancreas	1.9[b]	1.8[b]	1.8[b]	2.1[a]	0.08	0.272	0.073	0.006
Liver	19.7	23.3	22.6	25.5	1.62	0.126	0.056	0.835
Heart	5.5	5.8	5.7	5.9	0.20	0.501	0.187	0.926
Length (cm/kg BW)								
Duodenum	10.7	10.3	11.2	11.5	0.34	0.026	0.893	0.317
Jejunum	43.2	43.2	45.3	46.6	2.16	0.209	0.755	0.747
Ileum	11.9	12.0	12.9	12.5	0.93	0.428	0.902	0.812
Cecum (average of the two)	6.1	6.3	6.9	6.0	0.30	0.427	0.199	0.078
Colon	3.2	3.4	3.5	3.6	0.20	0.371	0.460	0.864
Total intestine	69.1	68.9	72.7	74.2	2.93	0.133	0.821	0.788

[1]Data are presented as least-square means and pooled SEM. $n = 7$ per FL group, RFI rank, and sex; except for $n = 8$ high RFI *ad libitum* females.

[2]RFI was calculated for the experimental period from 9 to 30 days post-hatch.

[3]FL, feed intake level.

[a,b,c]Different superscripts within a row indicate significant difference ($P \leq 0.05$).
