# Supplementary material for: Feed Restriction Modifies Intestinal Microbiota-Host Mucosal Networking in Chickens Divergent in Residual Feed Intake
Source: mSystems. 2019 Jan 29;4(1):e00261-18. doi: 10.1128/mSystems.00261-18 (PMC6351724; doi:10.1128/mSystems.00261-18)
Supplement: TABLE S5 [file mSystems.00261-18-st005.pdf]

TABLE S5 Differences in intestinal histo-morphology of low and high residual feed intake (RFI) broiler chickens fed either *ad libitum* or restrictively[1,2]

	Ad libitum feeding		Restrictive feeding			*P* value		
Parameter	low RFI	high RFI	low RFI	high RFI	SEM	FL[3]	RFI	FL × RFI
Jejunum								
Villus height (µm)	701	713	683	700	22.2	0.490	0.535	0.911
Villus width (µm)	93	98	101	100	4.0	0.212	0.572	0.444
Crypt depth (µm)	77	76	73	75	2.6	0.305	0.985	0.554
Villus height:crypt depth	9.1	9.6	9.5	9.4	0.35	0.757	0.668	0.463
Circular muscle (µm)	102	93	101	95	6.1	0.940	0.207	0.786
Longitudinal muscle (µm)	37	37	36	34	1.9	0.263	0.544	0.823
Goblet cells (counts/250 µm)	12.1	12.5	12.9	11.0	0.81	0.685	0.356	0.156
Lymphocytes (counts/400 µm)	7.0	7.3	6.0	6.4	0.62	0.149	0.600	0.983
Ileum								
Villus height (µm)	485	522	518	531	23.3	0.385	0.286	0.615
Villus width (µm)	98	95	92	99	3.6	0.817	0.497	0.154
Crypt depth (µm)	74	82	78	75	3.1	0.612	0.445	0.126
Villus height:crypt depth	6.6	6.4	6.9	7.1	0.31	0.148	0.992	0.495
Circular muscle (µm)	112	139	144	133	11.7	0.272	0.504	0.108
Longitudinal muscle (µm)	37	46	45	42	3.4	0.556	0.287	0.073
Goblet cells (counts/250 µm)	14.3	15.2	12.1	14.0	1.01	0.105	0.161	0.650
Lymphocytes (counts/400 µm)	6.3	6.3	5.2	5.2	0.60	0.068	0.979	0.927
Cecum								
Crypt depth (µm)	187	210	196	235	10.6	0.109	0.005	0.467
Circular muscle (µm)	240	252	243	272	16.8	0.500	0.232	0.612
Longitudinal muscle (µm)	60	68	59	67	4.2	0.794	0.061	0.957
Goblet cells (counts/250 µm)	4.2	5.0	3.3	5.0	0.46	0.368	0.008	0.319
Lymphocytes (counts/400 µm)	1.4	1.4	1.0	1.2	0.16	0.063	0.768	0.616

[1]Data are presented as least-square means and pooled SEM. n = 7 per FL group, RFI rank, and sex; except for n = 8 high RFI *ad libitum* females.

[2]RFI was calculated for the experimental period from 9 to 30 days post-hatch.

[3]FL, feed intake level.
