# Supplementary material for: Feed Restriction Modifies Intestinal Microbiota-Host Mucosal Networking in Chickens Divergent in Residual Feed Intake
Source: mSystems. 2019 Jan 29;4(1):e00261-18. doi: 10.1128/mSystems.00261-18 (PMC6351724; doi:10.1128/mSystems.00261-18)
Supplement: TABLE S6 [file mSystems.00261-18-st006.pdf]

TABLE S6 Differences in expression levels of nutrient transporter, barrier and innate immune genes at the jejunal mucosa of low and high residual feed intake (RFI) broiler chickens fed either *ad libitum* or restrictively[1,2]

	Ad libitum feeding		Restrictive feeding			*P* value		
Gene of interest	low RFI	high RFI	low RFI	high RFI	SEM	FL[3]	RFI	FL × RFI
Jejunum								
IAP	0.507	0.539	0.554	0.488	0.0451	0.962	0.717	0.283
MUC1	0.337	0.226	0.209	0.335	0.0627	0.876	0.902	0.065
MUC2	0.423	0.407	0.432	0.452	0.0399	0.508	0.953	0.652
CLDN1	0.292	0.291	0.232	0.268	0.0336	0.224	0.603	0.572
CLDN5	0.711	0.386	0.962	0.238	0.0534	0.711	0.386	0.962
OCLN	0.732	0.763	0.710	0.738	0.0365	0.525	0.414	0.972
ZO1	0.532	0.494	0.510	0.493	0.0352	0.746	0.431	0.777
SGLT1	0.573	0.585	0.607	0.631	0.0401	0.319	0.656	0.885
GLUT2	0.408	0.470	0.376	0.433	0.0439	0.437	0.182	0.957
MCT1	0.357	0.439	0.452	0.478	0.0383	0.086	0.164	0.469
SMCT	0.068[b]	0.182[a]	0.209[a]	0.122[ab]	0.0466	0.391	0.772	0.035
IL1B	0.306	0.225	0.277	0.271	0.0632	0.890	0.493	0.553
IL6	0.390	0.361	0.344	0.376	0.0544	0.772	0.983	0.576
IL8	0.214	0.134	0.122	0.158	0.0399	0.399	0.579	0.149
IL10	0.201	0.202	0.211	0.223	0.0411	0.697	0.871	0.899
TGFB1	0.450	0.445	0.432	0.491	0.0560	0.799	0.635	0.569
NFKB	0.632	0.608	0.627	0.621	0.0386	0.911	0.695	0.820
TNFA	0.438	0.453	0.446	0.438	0.0342	0.926	0.917	0.745
TLR2	0.377	0.344	0.378	0.381	0.0662	0.768	0.824	0.787
TLR4	0.486	0.459	0.510	0.439	0.0486	0.971	0.317	0.644
Ceca								
IAP	0.524	0.517	0.514	0.477	0.0449	0.575	0.621	0.746
MUC1	0.299	0.245	0.258	0.280	0.0518	0.958	0.762	0.463
MUC2	0.206	0.262	0.280	0.197	0.0565	0.928	0.813	0.226
CLDN1	0.294	0.296	0.264	0.273	0.0347	0.449	0.868	0.920
CLDN5	0.337	0.316	0.280	0.304	0.0367	0.344	0.975	0.543
OCLN	0.610	0.604	0.607	0.549	0.0431	0.509	0.466	0.555
ZO1	0.702	0.673	0.636	0.637	0.0404	0.212	0.741	0.707
SGLT1	0.094	0.136	0.189	0.091	0.0382	0.515	0.460	0.074
GLUT2	0.145	0.043	0.103	0.089	0.0463	0.964	0.214	0.352
MCT1	0.438	0.471	0.430	0.609	0.0436	0.143	0.018	0.102
SMCT	0.007	0.066	0.127	0.009	0.0462	0.493	0.528	0.061
IL1B	0.312	0.295	0.220	0.339	0.0462	0.609	0.279	0.147
IL6	0.495	0.474	0.398	0.488	0.0540	0.447	0.528	0.310

IL8	0.366	0.311	0.228	0.314	0.0565	0.238	0.790	0.221
IL10	0.402	0.417	0.330	0.404	0.0653	0.520	0.501	0.654
TGFB1	0.539	0.506	0.445	0.561	0.0441	0.654	0.348	0.098
TNFA	0.373[b]	0.335[b]	0.368[b]	0.519[a]	0.0468	0.061	0.231	0.048
NFKB	0.615	0.495	0.649	0.617	0.0393	0.052	0.057	0.264
TLR2	0.509	0.458	0.474	0.479	0.0431	0.861	0.594	0.518
TLR4	0.510	0.471	0.552	0.500	0.0482	0.463	0.347	0.891

[1]Data are presented as least-square means and pooled SEM. n = 7 per FL group, RFI rank, and sex; except for n = 8 high RFI *ad libitum* females.

[2]RFI was calculated for the experimental period from 9 to 30 days post-hatch.

[3]FL, feed intake level.

[a,b,c]Different superscripts within a row indicate significant difference ($P \leq 0.05$).
