# Supplementary material for: Feed Restriction Modifies Intestinal Microbiota-Host Mucosal Networking in Chickens Divergent in Residual Feed Intake
Source: mSystems. 2019 Jan 29;4(1):e00261-18. doi: 10.1128/mSystems.00261-18 (PMC6351724; doi:10.1128/mSystems.00261-18)
Supplement: TABLE S7 [file mSystems.00261-18-st007.pdf]

TABLE S7 Dietary ingredients and chemical composition of diets (on as-fed basis)

Item	Starter<br>1 to 8 d post-hatch	Grower<br>9 to 20 d post-hatch	Finisher<br>21 to 30 d post-hatch
Ingredient (%)			
Corn	56.392	61.772	63.928
Soybean meal	37.032	31.432	29.068
Soybean oil	2.284	2.676	3.412
Monocalcium phosphate	1.460	1.412	1.240
Calcium carbonate	1.372	1.280	1.160
DL-Methionine	0.352	0.308	0.256
Natrium bicarbonate	0.204	0.212	0.136
Lysine-HCL 98	0.140	0.148	0.044
Salt	0.120	0.120	0.156
L-Threonine	0.044	0.040	0
Premix[1]	0.600	0.600	0.600
Analyzed chemical composition, g/kg DM			
DM	882	885	879
CP	228	207	194
Crude fat	51.1	47.5	61.0
Crude fiber	28.0	29.0	30.1
Crude ash	52.6	50.0	46.3
Starch	356	388	401
Sugar	55.0	49.9	45.4
Calcium	8.88	8.39	7.63
Phosphorus	6.90	7.05	6.66
ME, MJ/kg	12.0	12.0	12.4

[1]Provided per kilogram of complete starter diet (Garant - Tiernahrung GmbH, Pöchlarn, Austria): 12,500 IU of vitamin A, 5,000 IU of vitamin D_3, 75.0 mg of vitamin E, 6.0 mg of vitamin K3, 2.50 mg of vitamin B_1, 7.0 mg of vitamin B_2, 4.50 mg of vitamin B_6, 0.025 mg of vitamin B_{12}, 60.0 mg of nicotinic acid, 15.0 mg of pantothenic acid, 1.0 mg of folic acid, 0.25 mg of biotin, 1,582.065 mg of choline, 400.322 mg of choline chloride, 115.20 mg of betaine, 19.98 mg of ethoxyquin, 900.004 FTU of 6Phytase, 0.564 g of ß-glucan, 2.428 % of C 18:2, 2.804 % of polyenic acid, 30.059 mg of F-Xanto (total). Provided per kilogram of complete grower diet (Garant - Tiernahrung GmbH, Pöchlarn, Austria): 12,500 IU of vitamin A, 5,000 IU of vitamin D_3, 75.0 mg of vitamin E, 6.0 mg of vitamin K3, 2.50 mg of vitamin B_1, 7.0 mg of vitamin B_2, 4.50 mg of vitamin B_6, 0.025 mg of vitamin B_{12}, 60.0 mg of nicotinic acid, 15.0 mg of pantothenic acid, 1.0 mg of folic acid, 0.25 mg of biotin, 1,457.765 mg of choline, 400.322

mg of choline chloride, 115.20 mg of betaine, 19.98 mg of ethoxyquin, 900.004 FTU of 6Phytase, 0.618 g of ß-glucan, 2.692 % of C 18:2, 3.103% of polyenic acid, 30.866 mg of F-Xanto (total). Provided per kilogram of complete finisher diet (Garant - Tiernahrung GmbH, Pöchlarn, Austria): 5,000 IU of vitamin D_3, 75.0 mg of vitamin E, 6.0 mg of vitamin K3, 2.50 mg of vitamin B_1, 7.0 mg of vitamin B_2, 4.5 mg of vitamin B_6, 0.025 mg of vitamin B_{12}, 60.0 mg of nicotinic acid, 15.0 mg of pantothenic acid, 1.0 mg of folic acid, 0.25 mg of biotin, 1,404.717 mg of choline, 400.322 mg of choline chloride, 115.20 mg of betaine, 19.98 mg of ethoxyquin, 900.004 FTU of 6Phytase, 0.639 g of ß-glucan, 3.098 % of C 18:2, 3.572 % of polyenic acid, 31.189 mg of F-Xanto (total).
