# Supplementary material for: Feed Restriction Modifies Intestinal Microbiota-Host Mucosal Networking in Chickens Divergent in Residual Feed Intake
Source: mSystems. 2019 Jan 29;4(1):e00261-18. doi: 10.1128/mSystems.00261-18 (PMC6351724; doi:10.1128/mSystems.00261-18)
Supplement: TABLE S8 [file mSystems.00261-18-st008.pdf]

TABLE S8 Oligonucleotide nucleotide primers used for the gene expression experiment

Gene symbol[1]	Accession number[2]	Forward primer (5'-3')	Reverse primer (5'-3')	Amplicon size (bp)	E (%)[3]	R^2	Ref.[4]
Housekeeping genes							1
ACTB	NM_205518.1	GAGAAATTGTGCGTGACATCA	CCTGAACCTCTCATTGCCA	152	100.2	0.99	1
B2M	NM_001001750.1	ACTTCACACCCAGCAGCG	GGCACAGCTCAGAACTCGG	103	102.5	0.99	1
GAPDH	NM_204305.1	TATCTTCCAGGAGCGTGACC	TCTCCATGGTGGTGAAGACA	95	92.5	1.00	1
HPRT1	NM_204848.1	GATGAACAAGGTTACGACCTGGA	TATAGCCACCCTTGAGTACACAG AG	181	94.7	0.99	1
SNRPD3	NM_001007838.1	CCAGATATGTTGAAGAACGCTCCT	GTTGCCACGACCCATACCA	141	104.6	0.98	1
Target genes							
OCLN	NM_205128.1	CAGCACCTACCTCAACCAGTACAT	AGGCAGAGCAGGATGACGAT	102	107.4	0.99	1
ZO1	XM_015278975.1, XM_015278976.1, XM_015278977.1, XM_015278978.1, XM_015278979.1, XM_015278980.1, XM_015278981.1,	CAACTGGTGTGGGTTTCTGAA	TCACTACCAGGAGCTGAGAGGTA A	101	98.7	0.99	1
CLDN1	NM_001013611.2	CCAGGTGAAGAAGATGCGGA	GGTGTGAAAGGGTCATAGAAGGC	129	104.4	0.99	1
CLDN5	NM_204201.1	CAGGTCGCCAGAGATACAGG	GAAGCCTCTCATAGCCTAAGCAT C	157	99	0.99	1
IAP	XM_015291488.1, XM_015291489.1	CCACTCGCATGTCTTCACCTT	CATTGCCGTAGAGGATGCTG	116	91.1	1.00	1
TLR2	NM_001161650.1, NM_204278.1	GGGCACAGGTTGGGAGTG	CCAACGACCACCAGGATGA	112	104.4	0.99	1
TLR4	NM_001030693.1	TGCAGTTTCTGGATCTTTCAAG	GCGACGTTAAGCCATGGAA	144	107.0	0.99	1
TNFA	AY765397.1	TCGGGAGTGGGCTTTAAGAAG	GAAAGCCACTAGGAGCAGACA	112	104.7	0.99	1
IL1B	NM_204524.1	GGGCATCAAGGGCTACAA	CTGTCCAGGCGGTAGAAGAT	88	96.2	0.99	1
IL6	JN639847.1, HM179640.1, AJ250838.2, NM_204628.1	GAGGGCCGTTCGCTATTTG	ATTGTGCCCGAACTAAAACATTC	67	108.4	0.98	2

IL8	HM179639.1	TCCTGGTTTCAGCTGCTCTGT	CGCAGCTCATTCCCCATCT	61	113.1	0.97	2
IL10	EF554720.1	GCTGAGGGTGAAGTTTGAGG	AGACTGGCAGCCAAAGGTC	121	90.3	0.99	3
TGFB1	NM_001318456.1	CGGCCGACGATGAGTGGCTC	CGGGGCCCATCTCACAGGGA	120	99.3	0.99	2
NFKB	NM_205134.1	CCATGGTAACTCGGACAGGG	GCTGTTCGTAGTGGTAAGTCTGA	193	98.9	0.99	-
MUC1	XM_015279046.1, XM_015279045.1	GGGAATCTGTGGCCTGTTGA	TTCTCAGCATCTCTCCCCCA	83	93.2	0.99	-
MUC2	NM_001318434.1	GCCTGCCCAGGAAATCAAG	CGACAAGTTTGCTGGCACAT	59	99.3	0.99	2
SLC16A1 (MCT1)	NM_001006323.1, XM_015298930.1	CTTTGGCTGGCTTAGCTCG	TTGTAGTCACCATACATGTCGTTG A	162	93.6	0.99	1
SLC5A12 (SMCT)	XM_416173.5	GGCTTCAGCGTTTGGGACTA	TGCAGAAGATGGCACCGTAG	235	90.6	0.98	-
SLC5A1 (SGLT1)	NM_001293240.1	CAATGCTGCGGACATCTCTGTT	TGTTCCTGCTATGCCCACAAA	199	94.3	0.99	-
SLC2A2 (GLUT2)	NM_207178.1	CGGCTGGGGAGGGTGAAAGC	CCCGCCCAGCGATGATGAGG	106	92.2	0.99	4

[1] *ACTB*, *Gallus gallus* actin, beta; *B2M*, *Gallus gallus* beta-2-microglobulin; *CLDN1*, *Gallus gallus* claudin 1; *CLDN5*, *Gallus gallus* claudin 5; *GAPDH*, *Gallus gallus* glyceraldehyde-3-phosphate dehydrogenase; *HPRT1*, *Gallus gallus* hypoxanthine phosphoribosyltransferase 1; *IL1B*, *Gallus gallus* interleukin 1 beta; *IL6*, *Gallus gallus* interleukin 6; *IL8*, *Gallus gallus* interleukin 8; *IL10*, *Gallus gallus* interleukin 10; *MUC1*, *Gallus gallus* mucin 1; *MUC2*, *Gallus gallus* mucin 2; *NFKB*, *Gallus gallus* nuclear factor kappa B; *OCLDN*, *Gallus gallus* occludin; *SNRPD3*, *Gallus gallus* small nuclear ribonucleoprotein D3 polypeptide; *TGFB1*, *Gallus gallus* transforming growth factor beta 1; *TLR2*, *Gallus gallus* toll-like receptor 2; *TLR4*, *Gallus gallus* toll-like receptor 4; *TNFA*, *Gallus gallus* lipopolysaccharide-induced tumor necrosis factor-alpha factor; *ZO1*, *Gallus gallus* zona occludens 1.

[2] National Center for Biotechnology Information (NCBI) Entrez Gene (http://www.ncbi.nlm.nih.gov/sites/entrez?db=gene).

[3] E, PCR efficiency: $E = (10^{(-1/slope)}-1)\times100$.

[4] Ref., references for oligonucleotide primer sequences.

References

1. Metzler-Zebeli BU, Magowan E, Hollmann M, Ball MEE, Molnár A, Witter K, Ertl R, Hawken RJ, Lawlor PG, O’Connell NE, Aschenbach J, Zebeli Q. 2018.

Differences in intestinal size, structure, and function contributing to feed efficiency in broiler chickens reared at geographically distant locations. Poult Sci 97:578–591. https://doi.org/10.3382/ps/pex332.

2. Chen J, Tellez G, Richards JD, Escobar J. 2015. Identification of potential biomarkers for gut barrier failure in broiler chickens. Front Vet Sci 2:14. https://doi.org/10.3389/fvets.2015.00014.

3. Grenier B, Schwartz-Zimmermann HE, Caha S, Moll WD, Schatzmayr G, Applegate TJ. 2015. Dose-dependent effects on sphingoid bases and cytokines in chickens fed diets prepared with *Fusarium verticillioides* culture material containing fumonisins. Toxins (Basel) 7:1253–1272. https://doi.org/10.3390/toxins7041253.

4. Franssens L, Lesuisse J, Wang Y, Willems E, Willemsen H, Koppenol A, Guo X, Buyse J, Decuypere E, Everaert N. 2016. The effect of insulin on plasma glucose concentrations, expression of hepatic glucose transporters and key gluconeogenic enzymes during the perinatal period in broiler chickens. Gen Comp Endocrinol 232:67-75. https://doi.org/10.1016/j.ygcen.2015.12.026.
